# Berberine suppressed sarcopenia insulin resistance through SIRT1-mediated mitophagy

**DOI:** 10.1515/biol-2022-0648

**Published:** 2023-07-17

**Authors:** Xiaojuan Su, Danqi Yang, Yu Hu, Ying Yuan, Le Song

**Affiliations:** Department of Geriatric, Zhongshan Hospital (Xiamen), Fudan University, Xiamen 361015, China; Department of Geriatric, Zhongshan Hospital, Fudan University, No. 180, Fenglin Road, Xuhui District, Shanghai 200032, China

**Keywords:** berberine, sarcopenia, insulin resistance, SIRT1-mediated mitophagy

## Abstract

Abnormal mitochondrial function resulting in inadequate energy supply leads to sarcopenia and IR, suggesting that maintaining mitochondrial homeostasis by regulating mitophagy may be a promising strategy for sarcopenia IR therapy. Herein, we constructed sarcopenia mice model, which was treated with berberine and/or SIRT1/mitophagy inhibitors, and the activity of SIRT1/mitophagy signaling pathway was identified. Then, muscle tissue, blood biochemical index, inflammatory factors, GTT, and ITT were detected. We found that berberine treatment increased the body weight and alleviated d-galactose-induced weight loss in mice. SIRT1/mitophagy inhibitors suppressed the effects of berberine in the treatment of sarcopenia. The effect of berberine on the increase of muscle tissue, improving metabolic disorders, reducing the expression of inflammatory factors, and suppressing sarcopenia insulin resistance (IR) were reversed by SIRT1/mitophagy inhibitors. Our study establishes proof-of-concept to distinct the effect of berberine in sarcopenia IR, and provides strong evidence to support the hypothesis that berberine-induced SIRT1 triggers mitochondrial autophagy pathway and suppresses IR in sarcopenia.

## 1 Introduction

As an essential energy generator for tissue homeostasis, the quality and quantity of mitochondria were strictly controlled [1]. Mitophagy is an evolutionarily conserved cellular process which removes dysfunctional or excess mitochondria, thereby fine-tuning mitochondrial number and maintaining energy metabolism [2]. Mitophagy mediated by PINK1 and Parkin is a common mitochondrial quality control pathway. PINK1 and Parkin recruit damaged mitochondria and autophagosomes and then fuse with lysosomes to form autophagosomes and degrade mitochondria [3]. Therefore, the study of mitophagy regulated by which substances under pathological conditions is a key scientific questions that need to be answered urgently.

Sarcopenia is a syndrome caused by continuous loss of skeletal muscle mass, strength, and function [4]. Skeletal muscle is one of the mainly target of insulin and responsible for most glucose metabolism. Therefore, skeletal muscle loss may produce insulin resistance (IR), which leads to a large number of systemic inflammations and metabolic disorders [5]. In addition, reports showed that sarcopenia increases the incidence of type 2 diabetes [6]. Multiple studies have noted that sarcopenia and IR are related to mitochondria function abnormalities [7]. Abnormal mitochondrial function resulting in inadequate energy supply leads to sarcopenia and IR, suggesting that maintaining mitochondrial homeostasis by regulating mitophagy may be a promising strategy for sarcopenia IR therapy.

Berberine is the main bioactive component of Coptis which improves sensitivity of insulin and ameliorates metabolic disorders [8]. Berberine regulates IR of skeletal muscle and tissue inflammation by up-regulating expression level of GLUT4 and increasing skeletal muscle glucose uptake, accompanied by an increase in skeletal muscle fiber number [9]. Therefore, berberine plays an important role in skeletal muscle metabolism and insulin sensitivity. However, the relationship between mitophagy and berberine remains unclear.

## 2 Methods

### 2.1 Model construction of sarcopenia

The mice were divided into five groups (blank group, model group, treatment group, SIRT1 inhibition group, and mitochondrial autophagy inhibition group, *n* = 5). Model construction of sarcopenia was performed as described previously [10]. Except the blank group, the other four groups were given d-galactose (Med Chem Express, USA; 400 mg/kg, prepared with 0.9% normal saline) by intrabitoneal injection, once a day for 60 days.


**Ethical approval:** The research related to animal use has been complied with all the relevant national regulations and institutional policies for the care and use of animals, and has been approved by the Ethics Committee of Zhongshan Hospital, Fudan University (KZ0020).

### 2.2 Drug administration and tissue sample collection

The treatment group was given berberine (50 mg/kg, Med Chem Express, USA), intragastric administration once a day for 30 days. In addition to berberine, SIRT1 inhibition group was also given selisistat (20 mg/kg, Med Chem Express, USA) and was administered by intrabitoneal injection once every 3 days for 30 days. In addition to berberine, the mitochondria autophagy inhibition group was additionally given U0126-EtOH (10 mg/kg, Med Chem Express, USA) by intrabitoneal injection once every 3 days for 30 days. The mice were sacrificed to collect blood, gastrocnemius muscle, subcutaneous fat, visceral fat, and brown fat for subsequent use.

### 2.3 Biochemical index detection of serum

The blood was let to stand for about 30 min, and the serum was completely collected by centrifugation at 3,000 rpm/min at 4℃ for 10 min. The contents of blood glucose, insulin, triglycerides, cholesterol, low density lipoprotein cholesterol, high density lipoprotein cholesterol, free fatty acids, and C-reactive protein were detected.

### 2.4 Glucose tolerance test (GTT)

All groups of mice were fasted 16 h before the experiment and water intake during the fasting period was kept normal. Each mouse was weighed and labeled. The tail end of mouse was cut off by 1–2 mm and gently squeezed. After the blood was enriched into a drop, it was evenly smeared on the blood glucose test paper and recorded as the blood glucose value of 0 min. After 30 min, the glucose solution prepared with 0.9% saline solution was given intragastrically at 1 g/kg. The blood glucose was measured at 15, 30, 60, 90, and 120 min, respectively.

### 2.5 Insulin tolerance test (ITT)

All groups of mice were fasted 4 h before the experiment and water intake during the fasting period was kept normal. Each mouse was weighed and labeled. The tail end of mouse was cut off by 1–2 mm, and the tail was gently squeezed. After the blood was enriched into a drop, it was evenly smeared on the blood glucose test paper and recorded as the blood glucose value of 0 min. After 30 min, the insulin was given as intraperitoneal injection at 0.75 U/kg. The blood glucose was measured at 15, 30, 60, 90, and 120 min, respectively.

### 2.6 ELISA

ELISA assay was performed using ELISA kit (Wellbio, China). The sample diluent, standard, and serum samples were added to the enzyme-labeled well, 50 µL per well. Coated and incubated at 37℃ for 40 min. Add 350 µL washing buffer to each well, shake for 2 min. Biotinylated antibody diluent was added to the blank well, and 100 µL biotinylated antibody working solution was added to the other wells. The mixture was coated and incubated at 37℃ for 30 min. Add 350 µL washing buffer to each well, shake for 2 min. Each well was added with SABC working solution and incubated at 37℃ for 20 min. Then added 350 µL washing liquid to each well and shaken for 2 min. Added TMB mixture to each well and incubated at 37℃ for 10 min. Added 100 µL termination solution to each well and absorbance was measured at 450 nm with enzyme micro-plate reader.

### 2.7 Quantitative real-time PCR

RNA was extracted from gastrocnemius muscle, subcutaneous fat, visceral fat, and brown adipose tissue. Quantitative real-time PCR was performed after reverse transcription. Primer design is as follows (Jinweizhi Biotechnology, China): 5′-GGATGATATGACGCTGTGGC-3′ (forward) and 5′-ACAGGAGACAGAAACCCCAG-3′ (reverse) for SIRT1; 5′-CGAGCATCTTCTAGCCCTGA-3′ (forward) and 5′-TCCAGGAAGAGAGGAGGAGG-3′ (reverse) for PINK1; 5′-TCAAGAAGACCACCAAGCCT-3′ (forward) and 5′-AACCAGTGATCTCCCATGCA-3′ (reverse) for Parkin; 5′-TCAAGATAATCAGACGGCGC-3′ (forward) and 5′-TTGCTGTCCCGAATGTCTCC-3′ (reverse) for LC3; 5′-TCAAGAAGACCACCAAGCCT-3′ (forward) and 5′-AACCAGTGATCTCCCATGCA-3′ (reverse) for Parkin; 5′-TCTCCTGCGACTTCAACA-3′ (forward) and 5′-TGTAGCCGTATTCATTGTCA-3′ (reverse) for GAPDH. The relative expression of target gene (RQ) was calculated by comparing 2^−δδCt^ according to previous study [11].

### 2.8 Hematoxylin–eosin staining

Paraffin sections were baked at 60℃ for 1 h. Gradient dewaxing is followed by gradient rehydration. The tissue sections were immersed in hematoxylin solution and stained for 10 min. The sections were rinsed with water to remove unbound dyes. The sections were immersed in hydrochloric acid ethanol ultra-rapid differentiation solution for 3 s, and then soaked in PBS for 10 min, repeated three times. The sections were immersed in eosin solution for 5 min and rinsed with water to remove unbound dyes. The sections were dried at 60℃ and sealed with a drop of neutral gum.

### 2.9 Statistical analysis

All the results were analyzed by the software GraphPad Prism 7.0 and SPSS 16.0, and were presented as the mean  ±  SD. Beyond that, the comparisons among the four groups were performed by one-way ANOVA. *P*  <  0.05 was considered as statistically significant.

## 3 Results

### 3.1 Berberine alleviated sarcopenia through SIRT1/mitochondrial autophagy pathway

**Figure 1:**
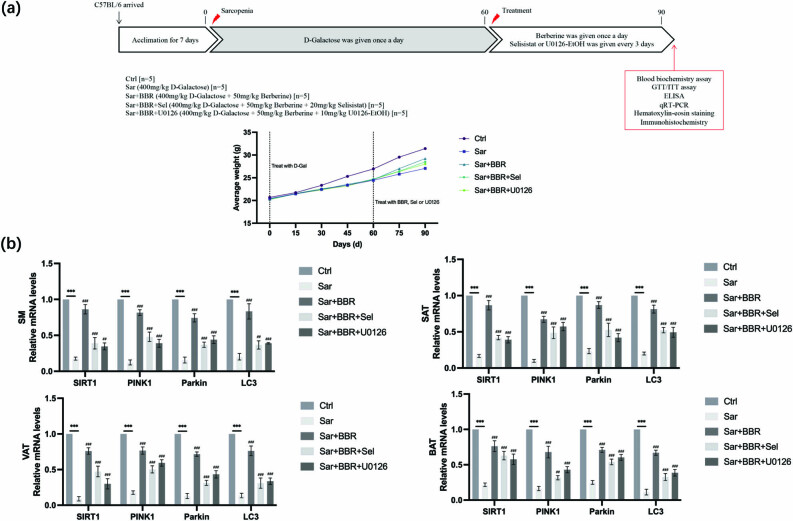
Identification of berberine as an inducer of SIRT1 mitochondrial autophagy-pathway in sarcopenia. (a) Anti-sarcopenia activity of berberine in sarcopenia model mice. Mice were treated with d-galactose (400 mg/kg orally) by intrabitoneal injection or control (0.9% normal saline) once a day for 60 days. Then, the mice were divided into five groups (blank group, model group, treatment group, SIRT1 inhibition group, and mitochondrial autophagy inhibition group, *n*  = 5). The treatment group was given berberine (50 mg/kg) by intragastric administration once a day for 30 days. In addition to berberine, SIRT1 inhibition group was also given selisistat (20 mg/kg) by intraperitoneal injection once every 3 days. In addition to berberine, the mitochondria autophagy inhibition group was additionally given U0126-EtOH (10 mg/kg) by intrabitoneal injection once every 3 days. The weight of mice in each group was determined once every 15 days. (b) Quantitative real-time PCR was performed to verify the role of SIRT1/mitophagy signaling pathway in berberine treating sarcopenia. The expression levels of SIRT1 and mitochondria autophagy related molecules (PINK1, Parkin, LC3II/I) in tissues were detected. Representative results were performed from independent experiments (*n*  = 5), error bars, SD. *P* values were indicated by a two-tailed unpaired Student’s *t*-test. **, *P* < 0.01; ***, *P* < 0.001; ##, *P* < 0.01; ###, *P* < 0.001.

To investigate the effect of berberine on suppressing sarcopenia, we constructed sarcopenia model by giving d-galactose (Figure 1a). Long-term exposure to d-galactose decreased the weight of the mouse (Figure 1a). Results showed that berberine treatment increased the body weight and alleviated d-galactose-induced weight loss in mice. Pink1 and Parkin-mediated mitophagy was a common mitochondrial quality control pathway. During mitophagy, Pink1 and Parkin recruited damaged mitochondria and autophagosomes, and then fused with lysosomes to form autolysosomes to degrade mitochondria [12]. Therefore, mitophagy inhibitor was given to verify whether berberine regulated mitophagy to improve sarcopenia. Results showed that the effect of berberine on sarcopenia was inhibited after treatment with mitophagy inhibitor (Figure 1a). Previous studies showed that SIRT1 mediated the activation of mitophagy [13]. Activation of SIRT1/PGC-1 pathway triggered autophagy/mitochondria and attenuated oxidative damage in intestinal epithelial cells [14]. In our study, it was also observed that SIRT inhibitors also suppressed the effects of berberine in the treatment of sarcopenia (Figure 1a).

In addition, we further detected the SIRT1/mitophagy signaling pathway in gastrocnemius muscle, subcutaneous fat, visceral fat, and brown adipose tissue. The results showed that SIRT1/mitophagy signaling pathway was inhibited by d-galactose, which indicated that sarcopenia decreased the activity of SIRT1/mitophagy signaling pathway. However, after berberine treatment, SIRT1/mitophagy signaling pathway was activated (Figure 1b). These results suggested that berberine ameliorates sarcopenia through SIRT1/mitophagy pathway.

### 3.2 Berberine alleviated the loss of muscle tissue in sarcopenia mice

**Figure 2:**
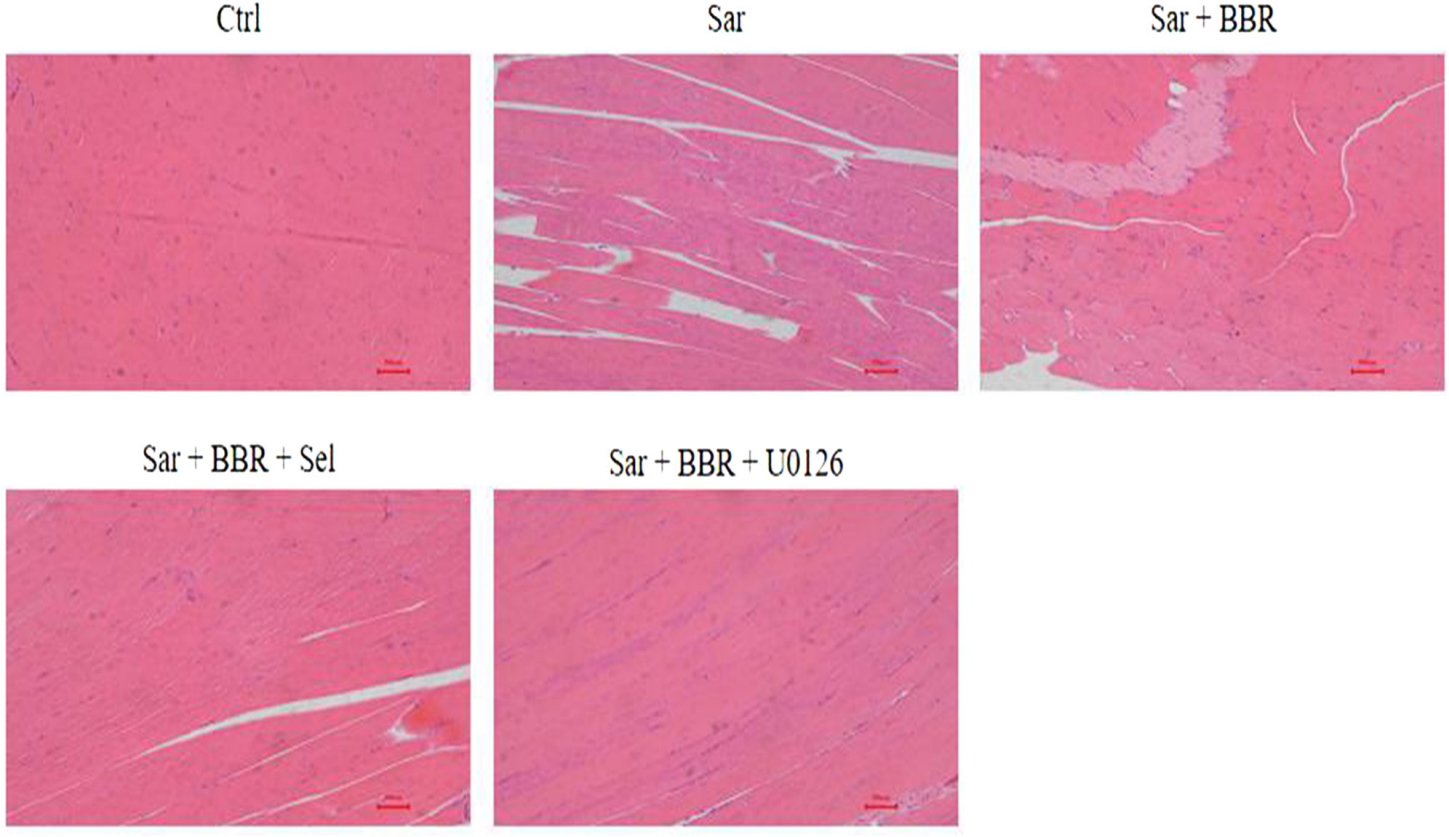
Muscle tissue significantly increased after treatment with berberine. Gastrocnemius tissue was observed by HE staining. Scale bar, 10 µm. Representative results were performed from independent experiments (*n*  = 5).

Loss of muscle tissue is the main character of sarcopenia [4], which was also found in d-galactose-treated mice in our study. Berberine significantly increased muscle tissue in d-galactose-treated mice. Mechanically, berberine alleviated muscle tissue loss in sarcopenia via SIRT1/mitophagy pathway. The effect of berberine on the increase of muscle tissue was reversed by SIRT1 and mitophagy inhibitor (Figure 2).

### 3.3 Berberine improved metabolic disorders associated with sarcopenia

**Figure 3:**
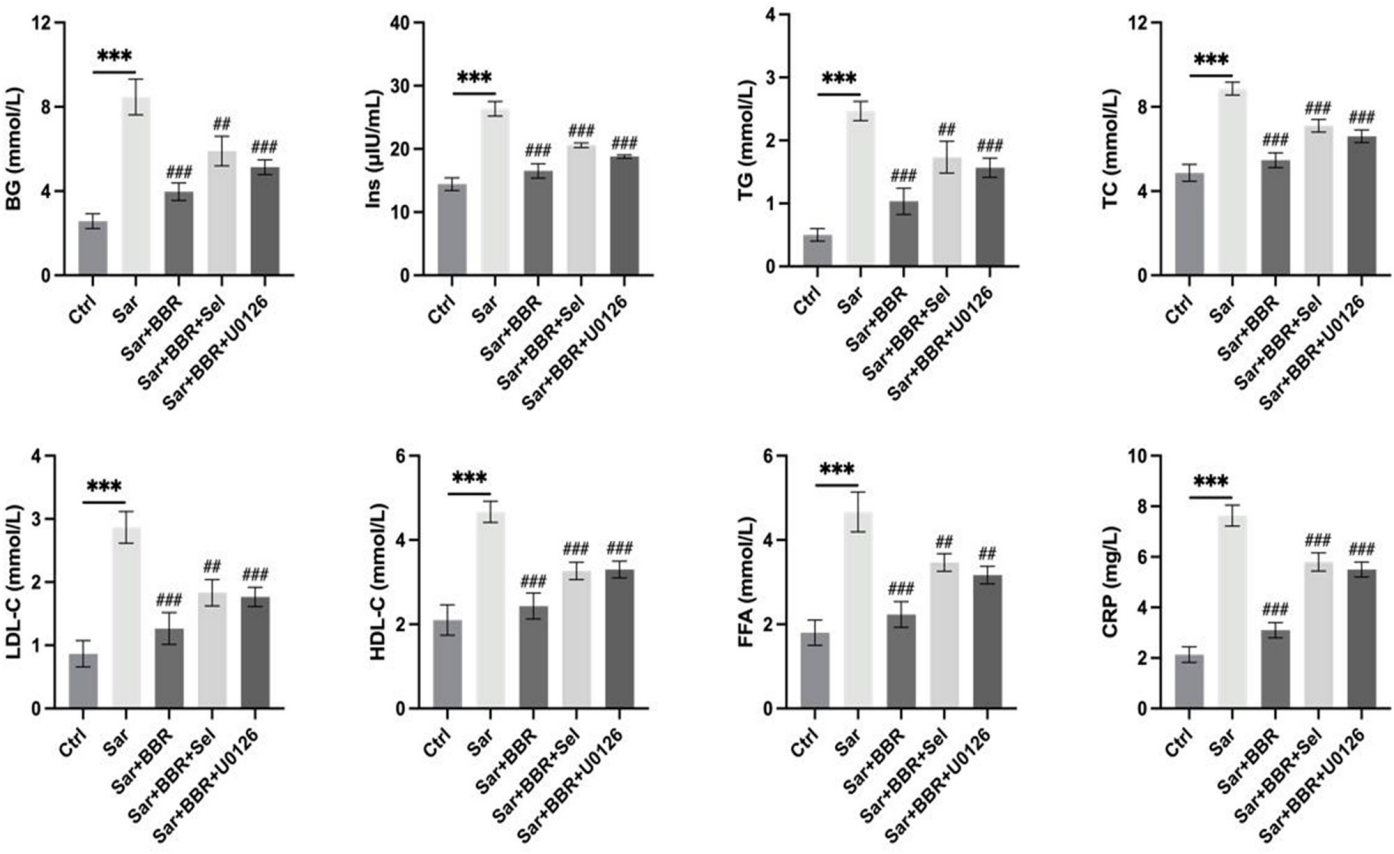
Effect of regulating metabolic disorders in sarcopenia of berberine was observed by blood biochemical index detection. Representative results were performed from independent experiments (*n*  = 5), error bars, SD. *P* values were indicated by a two-tailed unpaired Student’s *t*-test. ***, *P* < 0.001; ##, *P* < 0.01; ###, *P* < 0.001.

As an important target of insulin, skeletal muscle was responsible for most glucose metabolism in the body. Loss of skeletal muscle may produce IR, which leads to a large number of systemic inflammation and metabolic disorders [15]. In our study, metabolism was altered in mice treated with d-galactose, among which blood glucose, insulin, triglycerides, triglycerides, cholesterol, LDL cholesterol, HDL cholesterol, free fatty acids, and C-reactive protein were significantly up-regulated. Notably, the metabolism of mice tended to be the same as the control group after treatment with berberine. To further investigate the mechanism of how berberine regulated metabolic disorders in sarcopenia, blood biochemical index was detected after treatment of SIRT1 and mitochondrial autophagy inhibitors, while the effect of berberine on regulating metabolic disorders was inhibited by SIRT1 and mitochondrial autophagy inhibitors (Figure 3). In conclusion, berberine regulated sarcopenia related metabolic disorders, and its implied mechanism was activation of SIRT1/mitochondrial autophagy pathway.

### 3.4 Berberine reduced the expression of inflammatory factors in sarcopenia mice

**Figure 4:**
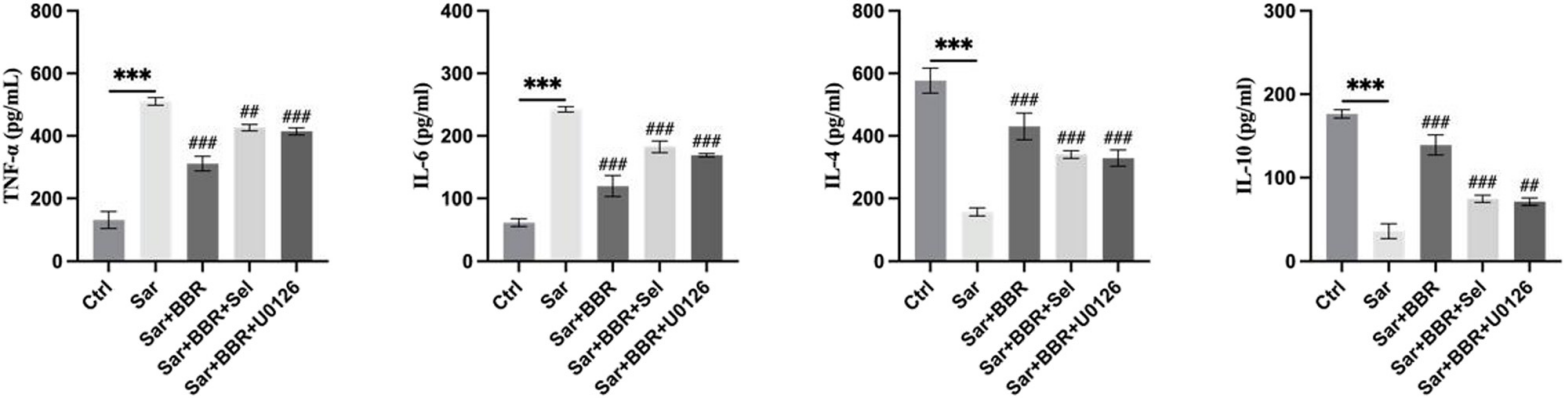
Berberine reduced the expression of inflammatory factors in sarcopenia mice. The levels of inflammatory factors (TNF-α, IL-6, IL-4, and IL-10) in tissues were detected by ELISA kit. Representative results were performed from independent experiments (*n*  = 5), error bars, SD. *P* values were indicated by a two-tailed unpaired Student’s *t*-test. ***, *P* < 0.001; ##, *P* < 0.01; ###, *P* < 0.001.

Our previous results revealed that the metabolic disorders of sarcopenia were decreased by berberine. However, whether berberine influenced the systemic inflammation of sarcopenia is unclear. Since berberine ameliorated tissue inflammation by up-regulating GLUT4 expression and increasing skeletal muscle glucose uptake, and was associated with an increase in skeletal muscle fiber number [16], we tested the possibility that berberine ameliorated tissue inflammation via SIRT1/mitochondrial autophagy pathway. The ELISA assay demonstrated that berberine down-regulated the expression of TNF-α and IL-6, as well as elevated the expression of IL-4 and IL-10. Moreover, treatment to SIRT1 and mitochondrial autophagy inhibitors rescued the effect of berberine reducing the expression of inflammatory factors (Figure 4). These results suggested that berberine suppressed systemic inflammation of sarcopenia through SIRT1/mitochondrial autophagy pathway.

### 3.5 Berberine exhibits significant effect on suppressing sarcopenia IR

**Figure 5:**
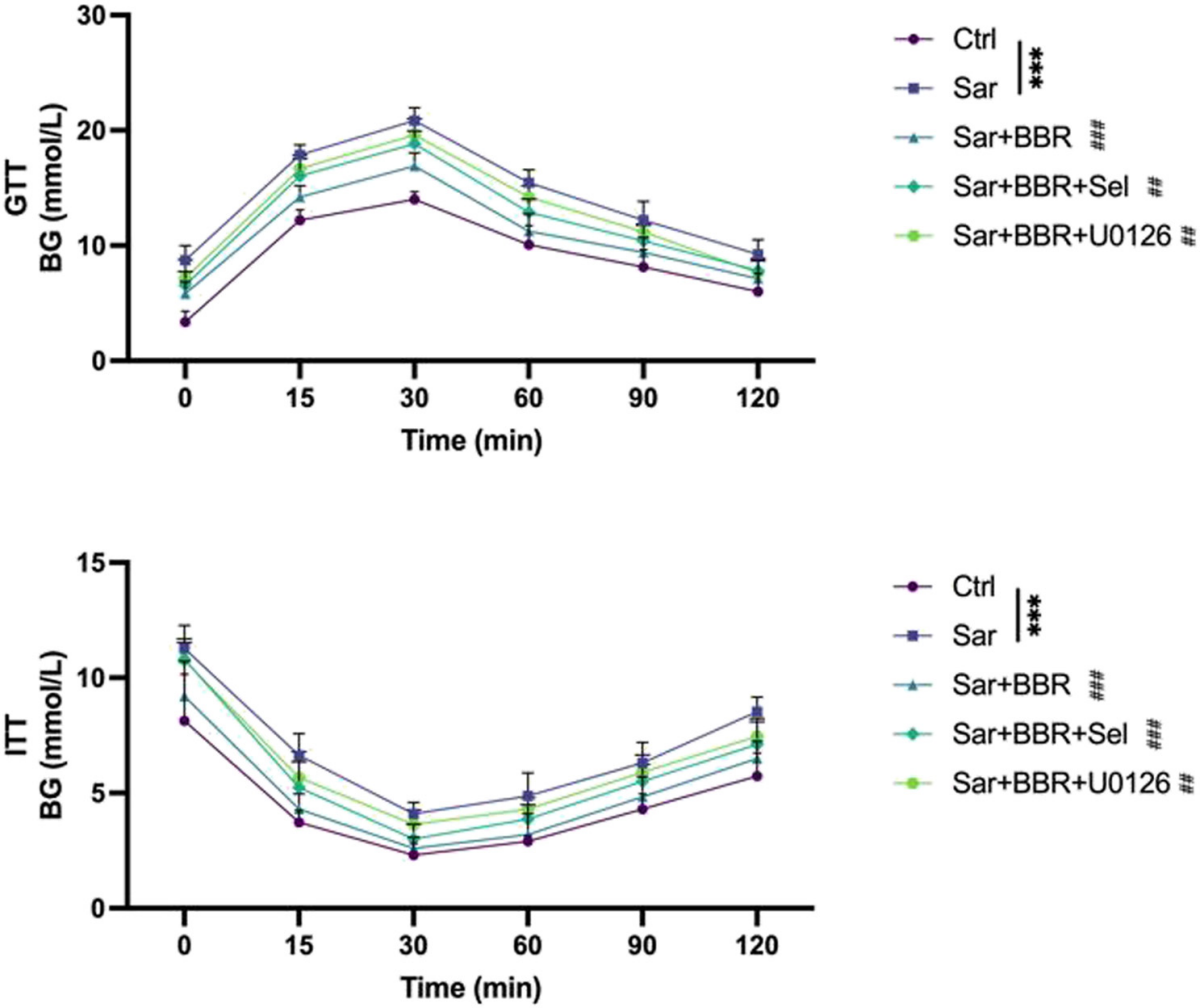
Berberine exhibits significant effect on suppressing sarcopenia IR. The levels of GTT and the ITT were measured by a glucose analyzer at 4, 8, 16, and 20 weeks, respectively. Representative results were performed from independent experiments (*n*  = 5), error bars, SD. *P* values were indicated by a two-tailed unpaired Student’s *t*-test. ***, *P* < 0.001; ##, *P* < 0.01; ###, *P* < 0.001.

Recent study revealed that sarcopenia increases the risk of type 2 diabetes [6]. In addition, many symptoms of metabolic syndrome such as obesity, hyperglycemia, and abnormal lipid metabolism are associated with sarcopenia [15]. We observed that d-galactose decreased the GTT and ITT of sarcopenia model mouse, while treatment with berberine increased the GTT and ITT of sarcopenia model mouse (Figure 5). To confirm this effect, the GTT and ITT of sarcopenia model mouse treated with berberine were determined after treatment to SIRT1 and mitochondrial autophagy inhibitors. Taken together, Berberine relieved IR in sarcopenia, and the relief of IR in sarcopenia by berberine depends on SIRT1/mitochondrial autophagy pathway.

## 4 Discussion

In this study, we identified berberine as a novel inducer of mitophagy, exhibiting a significant effect on sarcopenia IR. Berberine increases the SITRT1 activity and accelerates mitophagy by PINK1/Parkin pathway. We also show that berberine ameliorates sarcopenia IR mainly by inducing mitophagy. Thus, our results reveal the mechanism under the effect of berberine on sarcopenia IR via PINK1/Parkin-inducing mitophagy, which provides a new paradigm to develop promising therapeutic strategies for sarcopenia IR. It was revealed that berberine ameliorated IR in sarcopenia. The implied mechanism was through up-regulating the SIRT1/mitochondrial autophagy pathway.

Multiple studies have noted that sarcopenia increases the risk of type 2 diabetes [17]. Sarcopenia IR is harmful to the physical and mental health of patients, but the existing clinical prevention and treatment strategies are limited. In recent years, traditional Chinese medicine and its extracts have broad development and application value due to their advantages of low price, easy access to materials, and remarkable effects [18]. Berberine shows significant effect on improving insulin sensitivity and metabolic disorders [19]. Berberine also showed critical role in regulating abnormal bone metabolism [20]. Mechanically, berberine regulates skeletal muscle metabolism through various ways. For example, the metabolism of skeletal muscle cells is regulated through AMPK/SIRT1 and PCG-1α pathways [20]. Another study has shown that berberine suppresses skeletal muscle IR by down-regulating TLR4/IKKβ/NF-κB and PPARα signaling [21]. Therefore, berberine plays an important role in skeletal muscle metabolism and insulin sensitivity. It was also observed in our study that berberine alleviates the symptoms of IR in sarcopenia. However, the mechanism of berberine in ameliorating IR in sarcopenia is still unclear.

In view of this, this study identified a strategy for treating IR in sarcopenia through berberine. Preliminary experiment confirmed that the expression of SIRT1 was significantly up-regulated in skeletal muscle and adipose tissue of mice after the treatment of berberine. SIRT1 is a niacin amine adenine dinucleotide (NAD+) dependent histone deacetylase, which participates in physiological activities such as cell aging, apoptosis, and differentiation [22]. Previous studies have found that SIRT1 in vascular endothelial cells has antioxidant stress effects. Notably, berberine in adipose tissue inhibits tissue inflammation and insulin sensitivity by up-regulating SIRT1 signal [23,24]. In addition, berberine plays a protective role in brain trauma by regulating SIRT1 signal [25]. Interestingly, our results showed that berberine up-regulated the expression of SIRT, and SIRT inhibitors inhibited the effect of berberine, suggesting that berberine mainly inhibits sarcopenia by regulating SIRT1. According to the above information, the protective effect of berberine depends on SIRT1 signal. However, the downstream molecular mechanism of SIRT1 still needs to be further explored.

It was aware of that sarcopenia and insulin resistance were related to mitochondria function mitochondria [26]. Abnormal mitochondrial function results in insufficient energy supply and leads to sarcopenia and IR. Therefore, maintaining mitochondrial homeostasis represents a promising strategy for IR in sarcopenia. Mitophagy is an evolutionally conserved cellular process to clear dysfunctional or redundant mitochondria, thus fine-tuning mitochondrial number and maintaining energy metabolism [27,28]. Mitochondrial autophagy mediated by PINK1 and Parkin is a common mitochondrial quality control pathway [12,29]. Meanwhile, SIRT1 mediates the activation of mitochondrial autophagy [13]. SIRT1/PGC-1 pathway activates autophagy/mitochondria and weakens oxidative damage in intestinal epithelial cells [30]. MiR-34a/SIRT1 signaling protects cochlear hair cells from oxidative stress and delays age-related hearing loss by regulating mitochondrial autophagy and mitochondrial biogenesis [31]. At present, the role of mitochondrial autophagy in sarcopenia has been preliminarily confirmed [32]. There is also a certain correlation between mitochondrial autophagy and IR [33]. Results of our study also confirmed that after treatment of berberine in IR mice with sarcopenia, the expression level of mitochondrial autophagy related molecules (PINK1 and Parkin) was significantly up-regulated. Meanwhile, SIRT1-mediated mitochondrial autophagy activation in sarcopenia IR is the main mechanism by which berberine ameliorates IR in sarcopenia, and that mitochondrial autophagy may play an important role in IR in sarcopenia.

In summary, our study establishes proof-of-concept to distinct the effect of berberine in sarcopenia IR, and provides strong evidence to support the hypothesis that berberine-induced SIRT1 triggers mitochondrial autophagy pathway and suppresses IR in sarcopenia. Understanding the precise role of berberine in treating sarcopenia IR and the regulation of SIRT1/mitochondrial autophagy pathway will advance our knowledge of sarcopenia pathogenesis.
